# Security Concerns to Be Considered When Downloading Human Immunodeficiency Virus/Sexually Transmitted Disease Related Smartphone Applications

**DOI:** 10.2196/jmir.2650

**Published:** 2013-10-07

**Authors:** Savita Lorreta Brito-Mutunayagam, Imali Fernando

**Affiliations:** ^1^Chalmers Sexual Health CentreEdinburghUnited Kingdom

**Keywords:** HIV/STD, smartphone applications, security

“Mobile Phone Applications for the Care and Prevention of HIV and Other Sexually Transmitted Diseases: A Review” by Muessig et al is an excellent review that succinctly summarizes currently available mobile phone applications (apps) related to the prevention and care of human immunodeficiency virus (HIV) and other human immunodeficiency virus/sexually transmitted disease (STDs) [1]. The authors have comprehensively reviewed apps related to HIV/STDs and identified the need for health care professionals to work closely with app developers to provide accurate evidence based advice, and design effective risk reduction interventions.

While Muessig et al rightly raised concerns regarding the accuracy and reliability of the content of these apps, an important area not discussed in their review is the security of the apps. When downloading an app, the user is asked to authorize the “permissions” requested by the application. These “permissions” enable the optimum performance of the app on a smartphone [2]. There are over 100 different “ permissions” requested by smartphone applications. While some of the “permissions” requested are harmless, many raise serious concerns regarding the confidentiality and security of the apps requesting them [2].  These include permissions that request;

The above mentioned permissions that an app may require for optimum functioning involve access to and control of sensitive personal data. Applications often have legitimate reasons for accessing this sensitive and private data. Permission to obtain the exact GPS location of the app user is necessary if the app is designed to provide information on the nearest HIV/ STD testing center. If the app is designed as a personal assistant for those living with HIV, access to the user’s calendar is important to remind them of their next hospital appointment.

However, the concern arises when the app is not developed by a named professional health care body/organization and there is no assurance of confidentiality. Today’s smartphone applications often fail to provide users with visibility into where their private data is being stored and how it is being used.  There are often significant social implications associated with a diagnosis of HIV and the secure storage of their personal information is of immense importance to those living with the condition. Even individuals simply looking for information on the topic, or calculating their risk of contracting a STD after unprotected sexual intercourse, may be concerned if an unverified smartphone application had access to their personal information including precise location.

Muessig et al reviewed HIV and STD related apps that matched their search criteria in the Apple iTunes Store and the Android Google Play Store, as combined these two companies account for over 86% of the global app market [1]. In the android store this information is readily available within the app details.  Apple does not explicitly specify permissions required in the app details, but this information is available on download.  Apple states that all their apps are pre-screened prior to making them available for download. However, the recent controversy  surrounding Apple, for enabling the download of malicious apps that stole their users’ address books, show that this screening process is not infallible [3].

Providing HIV and STD prevention and care services via smartphone applications is an area of rapid and immense growth. If provided by trusted and professional organizations which guarantee the security of their users’ personal information, they can be a powerful and rapidly accessible resource. However, it is essential that users are aware of the  potential confidentiality and security breaches when downloading these apps. They must be encouraged to pay attention to the app developer in order to ascertain if it is a reputed body Furthermore, they should note the permissions requested by the apps and only proceed with the download if they are comfortable with these requests. Whilst more vigilance amongst app users is essential, it is also the responsibility of the companies that offer these apps to ensure their products are not malicious and employ the highest levels of data protection software.
